# In Situ Atomic‐Scale Investigation of Structural Evolution During Sodiation/Desodiation Processes in Na_3_V_2_(PO_4_)_3_‐Based All‐Solid‐State Sodium Batteries

**DOI:** 10.1002/advs.202301490

**Published:** 2023-09-06

**Authors:** Fang‐Chun Shen, Qianli Ma, Frank Tietz, Jui‐Cheng Kao, Chi‐Ting Huang, Rahmandhika Firdauzha Hary Hernandha, Chun‐Wei Huang, Yu‐Chieh Lo, Jeng‐Kuei Chang, Wen‐Wei Wu

**Affiliations:** ^1^ Department of Materials Science and Engineering National Yang Ming Chiao Tung University Hsinchu 30010 Taiwan; ^2^ Forschungszentrum Jülich GmbH Institute of Energy and Climate Research Materials Synthesis and Processing (IEK‐1) 52425 Jülich Germany; ^3^ Department of Materials Science and Engineering Feng Chia University No. 100, Wenhwa Rd., Seatwen Taichung 40724 Taiwan; ^4^ Center for the Intelligent Semiconductor Nano‐system Technology Research Hsinchu 30078 Taiwan

**Keywords:** all‐solid‐state, atomic‐scale, dynamic evolution, in situ TEM, Na_2_V_2_(PO_4_)_3_, Na_3_V_2_(PO_4_)_3_

## Abstract

Recently, all‐solid‐state sodium batteries (Na‐ASSBs) have received increased interest owing to their high safety and potential of high energy density. The potential of Na‐ASSBs based on sodium superionic conductor (NASICON)‐structured Na_3_V_2_(PO_4_)_3_(Na_3_VP) cathodes have been proven by their high capacity and a long cycling stability closely related to the microstructural evolution. However, the detailed kinetics of the electrochemical processes in the cathodes is still unclear. In this work, the sodiation/desodiation process of Na_3_VP is first investigated using in situ high‐resolution transmission electron microscopy (HRTEM). The intermediate Na_2_V_2_(PO_4_)_3_ (Na_2_VP) phase with the P2_1_/c space group, which would be inhibited by constant electron beam irradiation, is observed at the atomic scale. With the calculated volume change and the electrode–electrolyte interface after cycling, it can be concluded that the  Na_2_VP phase reduces the lattice mismatch between Na_3_VP and NaV_2_(PO_4_)_3_ (NaVP), preventing structural collapse. Based on the density functional theory calculation (DFT), the Na^+^ ion migrates more rapidly in the Na_2_VP structure, which facilitates the desodiation and sodiation processes. The formation of  Na_2_VP phase lowers the formation energy of NaVP. This study demonstrates the dynamic evolution of the Na_3_VP structure, paving the way for an in‐depth understanding of electrode materials for energy‐storage applications.

## Graphical abstract

The structural evolution of Na_3_VP is demonstrated with in situ transmission electron microscopy (TEM). Outside the in situ area, the intermediary Na_2_VP phase with the space group P2_1_/c is observed at atomic scale, which reduces the lattice mismatch between Na_3_VP and NaVP. The Na_2_VP phase lowers the formation energy of NaVP and possesses lower energy barrier for Na^+^ diffusion.

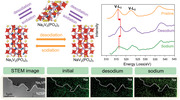

## 1 Introduction

Ever since Li*_x_* C_6_/organic electrolyte/Li_1−_ *_x_* CoO_2_ batteries were successfully commercialized by Sony Corporation in 1991, these lithium‐ion batteries (LIBs) have dominated the portable devices and consumer electronics market.^[^ ^1^, ^2^ ^]^ For the automotive sector, the development of LIBs with liquid electrolytes has stagnated owing to its flammable liquid electrolytes and insufficient energy density.^[^ ^3^, ^4^ ^]^ The ever‐increasing demand for energy storage systems has invigorated the need to seek alternatives with higher performance, enhanced safety, and increased economic efficiency compared to conventional LIBs. In this respect, all‐solid‐state batteries (ASSBs) are regarded as the potential solution. Although the phenomenon occurring in a liquid electrolyte system and in a solid‐state electrolyte system is identical, ASSBs can eliminate the safety concerns of traditional batteries by replacing flammable liquid electrolytes with solid electrolytes. Furthermore, solid electrolytes are more chemically and electrochemically stable than liquid electrolytes.^[^ ^5^ ^]^ In addition, batteries with solid‐state design also show better adaptability to temperature changes, and have the potential of achieving both higher energy and power densities.^[^ ^6^ ^]^

Li has been the preferred in battery chemistries in recent decades. However, the cost of future LIBs remains uncertain due to the increasing demand of LIBs and uneven geographical distribution of Li.^[^ ^7^, ^8^ ^]^ The search for alternative high‐energy batteries using abundant and low‐cost materials is imperative. Among the possible candidates, sodium‐ion batteries (SIBs) possess advantages such as abundant sodium supply, high energy storage capacity, and high electrochemical activity, which have attracted the attention of researchers.^[^ ^9^, ^10^, ^11^, ^12^, ^13^, ^14^ ^]^ Na_3_V_2_(PO_4_)_3_ (Na_3_VP) is a promising electrode material, which crystallizes in a NASICON structure.^[^ ^15^, ^16^, ^17^, ^18^ ^]^ It shows very promising capability in Na‐ASSBs. Having the same structure as the highly conductive solid electrolyte Na_3.4_Zr_2_Si_2.4_P_0.6_O_12_ (NZSP), Na_3_VP showed excellent chemical compatibility with NZSP and great cycling stability when used as the positive electrode material.

The structure of Na_3_VP is a 3D open framework composed of repeating lantern units, where two VO_6_ octahedra are joined together by three corner‐sharing PO_4_ tetrahedra. There are two different Na sites located in the framework: Na(1) and Na(2). The Na(1) sites are the octahedral sites between two VO_6_ octahedra, whereas the Na(2) sites are the tetrahedral sites between two adjacent PO_4_ tetrahedra along the *c*‐axis direction. The open diffusion paths and rigid structure in the polyanionic sublattice can be attributed to the strong covalent bonds between the oxygen atoms. The Na^+^ ions can reversibly diffuse through the interstitial sites without a significant volume change^[^ ^19^ ^]^ in the crystal structure, achieving high theoretical energy storage capacities and superior ionic mobility. Previous studies have demonstrated the high energy density, high rate, and long cycle life of Na_3_VP‐based sodium‐ion batteries, which are closely related to the Na_3_VP microstructure.^[^ ^20^, ^21^, ^22^ ^]^ However, the structural evolution of Na_3_VP during sodiation/desodiation remains ambiguous. Therefore, it is essential to investigate the Na^+^ ion insertion and extraction mechanism of Na_3_VP.

There have been growing interests in developing in situ techniques for battery studies, such as operando X‐ray diffraction (XRD) technique^[^ ^23^ ^]^ and in situ Raman methods.^[^ ^24^ ^]^ For example, the low strain of the Na_3_V_2_(PO_4_)_2_O_2_F lattice with a volumetric variation of only 2.56% during Na intercalation/extraction processes was disclosed by operando XRD.^[^ ^25^ ^]^ In situ Raman analysis revealed a highly reversible three‐phase transition as the sodium‐ion storage mechanism of Na_2_FeFe(CN)_6_ during sodiation/desodiation processes.^[^ ^26^ ^]^ Among various in situ techniques, in situ TEM possesses the capability of resolving the microstructural evolution and a providing diffraction information of the electrode materials. It can create a variety of external fields, including electric and thermal fields, and can observe the dynamic structure evolutions at the same time. Based on the advantages mentioned above, in situ transmission electron microscopy (TEM) has also been utilized to observe various reaction phenomena, such as catalytic phenomena,^[^ ^27^, ^28^ ^]^ electromigration,^[^ ^29^, ^30^ ^]^ and electrochemical reaction of batteries.^[^ ^31^, ^32^, ^33^ ^]^ In this work, an all‐solid‐state battery cell was prepared and the phase transition of Na_3_VP under charging/discharging was investigated using operando X‐ray diffractometer (XRD) analysis. Moreover, the structural evolution of Na_3_VP during sodiation/desodiation process was observed via in situ high‐resolution transmission electron microscopy (HRTEM) for the first time. The selected area electron diffraction (SAED) images of Na_3_VP demonstrated the evolution of lattice spacing, verifying the Na^+^ ions insertion/extraction during sodiation/desodiation process. Density functional theory (DFT) computations were employed to determine the formation energies of the cathode and the energy barrier for Na^+^ ions diffusing in the cathode structure. In‐depth information about the mechanism of the phase transition in the Na_3_VP–NaV_2_(PO_4_)_3_ (NaVP) system will be discussed in future research. This study unveils the structural evolution of Na_3_VP during the sodiation/desodiation process, providing fundamental information on Na_3_VP and laying the foundation for further research and future applications of Na‐ASSBs.

## 2 Results and Discussion

**Figure 1:**
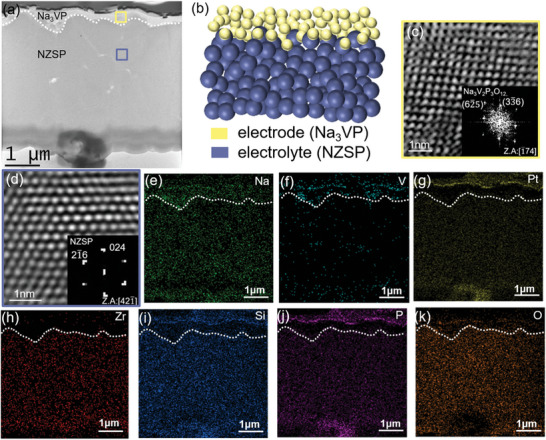
Pristine state of the sample observed using TEM and EDS. a) TEM image of the sample. b) Schematic diagram showing the Na_3_VP electrode and the NZSP electrolyte. c,d) HRTEM images of Na_3_VP and NZSP, respectively. EDS images of e) Na, f) V, g) Pt, h) Zr, i) Si, j) P, and k) O, respectively. The white dotted lines represent the interface between Na_3_VP and NZSP.

Figure S1 in the Supporting Information shows the as‐synthesized all‐solid‐state Na_3_VP/NZSP sample. The XRD spectra of the sample showed that the diffraction peaks of Na_3_VP and NZSP were both indexed to the NASICON structure with the R$\bar{3}$c space group (Figure S2, Supporting Information).^[^ ^34^, ^35^ ^]^Detailed information on the crystal structures of Na_3_VP and NZSP is provided in Figure S3 in the Supporting Information. The microstructure of the sample was investigated with TEM. The interface between Na_3_VP and NZSP was continuous, indicating good contact between the solid‐state electrode and electrolyte (as shown in **Figure** **1**a). An enlarged TEM image of the interface is shown in Figure S4 in the Supporting Information. The schematic diagram illustrates the sample comprising the Na_3_VP cathode and NZSP electrolyte (Figure 1b). The HRTEM images and corresponding diffraction patterns showed that both Na_3_VP (Figure 1c) and NZSP (Figure 1d) were crystalline with a rhombohedral structure. According to the EDS mapping data (Figure 1e–k), the distribution of V, Zr, and Si confirms the presence of Na_3_VP and NZSP. The aggregation of the reactant particles in the precursor solution during the fabrication of the sample may result in the nonuniformity of V in the Na_3_VP cathode.^[^ ^36^ ^]^Although the Si signal interferes with the SiN*_x_* membrane of the in situ TEM chip, the distribution of the Si signal in the NZSP still has a stronger contrast. The electrochemical measurements performed by our group previously demonstrated that Na_3_VP possessed stable performance with slow capacity fading (Figure S5, Supporting Information).^[^ ^35^ ^]^ At full desodiation, the value of the volume change of Na_3_VP is ≈8.8%, which is larger than that of Na_4_MnV(PO_4_)_3_ (8.68%),^[^ ^37^ ^]^ Na_4_MnCr(PO_4_)_3_ (7.7%),^[^ ^38^ ^]^ and Na_3.8_MnV_0.8_Zr_0.2_(PO_4_)_3_ (4.9%).^[^ ^39^ ^]^ The result is against previous study since the larger volume change usually leads to poor cyclability.^[^ ^40^ ^]^

**Figure 2:**
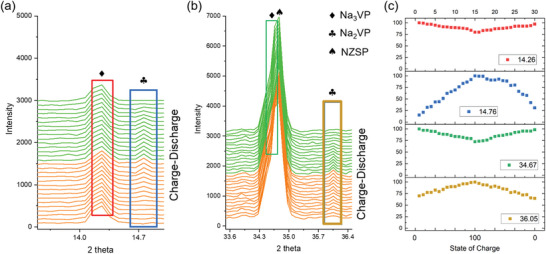
Selected in situ XRD patterns of Na_3_VP during first cycle with a voltage window of 2.7–4.0 V versus Na^+^/Na at the C‐rate of 0.4 C. The narrower 2*θ* range are a) 13.6°–15.0° and b) 33.6°–36.4°. c) The intensity evolution of the diffraction peaks at 14.26°, 14.76°, 34.67°, and 36.05°, respectively.

To investigate the structural evolution of Na_3_VP owing to its close relationship with the electrochemical performance, the operando XRD experiment was conducted in this work. Figure S6 in the Supporting Information shows the cell charged/discharged between 2.7 and 4 V and the selected operando XRD patterns is shown in **Figure** **2**. The Na_3_VP in R$\bar{3}$c space group was detected before cycling. During the charge process, the intensity of the diffraction peak of Na_3_VP phase at 14.26° gradually decreased, and a weak reflection at 14.76° started to appear close to the mid‐charge (as shown in Figure 2a). In the meanwhile, the diffraction peak of Na_3_VP phase at 34.67° gradually disappeared, accompanying a weak reflection at 36.05° (Figure 2b). During the discharge process, the peaks at 14.76° and 36.05° disappeared with an increased intensity of the diffraction peaks of Na_3_VP at 14.26° and 34.67°. Figure 2c illustrates the intensity evolution of the diffraction peaks during charging/discharging processes at 14.26°, 14.76°, 34.67°, and 36.05°, respectively. Previous study reported that the diffraction peaks of Na_2_VP phase appeared at 2*θ* ≈ 7.7° and 19.15° with the wavelength of incident X‐ray being 0.8266 Å.^[^ ^41^ ^]^ Based on the equation of Bragg's Law

(1) nλ=2dsinθ

where the diffraction order *n* and the lattice spacing *d* are constant for a specific plane, the diffraction peaks of Na_2_VP phase would appear at 14.37° and 36.12° with the wavelength of incident X‐ray being 1.54 Å in our work. The calculated 2*θ* (14.37° and 36.12°) are closed to the diffraction peaks (14.76° and 36.05°) appearing in the mid‐charge. It is suggested that the new reflections are attributed to the new intermediate Na_2_VP phase.

**Figure 3:**
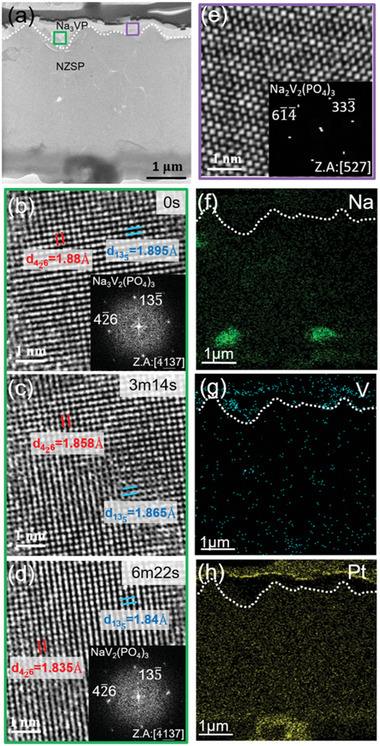
Desodiation of Na_3_VP cathode. a) TEM image of the sample after desodiation. The green and purple squares indicate the regions inside and outside the in situ area, respectively. b–d) HRTEM images demonstrating the desodiation process of Na_3_VP. e) HRTEM image of the intermediate Na_2_VP phase. EDS images of f) Na, g) V, and h) Pt, respectively. The white dotted lines represent the interfaces between Na_3_VP and NZSP.

To further understand the Na‐ion insertion/extraction mechanism, the desodiation process of the Na_3_VP cathode is investigated via in situ HRTEM. **Figure** **3**a shows the TEM image of the sample desodiated to 4 V. The initial lattice spacings were 1.88 and 1.895 Å, corresponding to the (4$\bar{2}$6) and (1$\bar{3}$5) planes of the Na_3_VP crystal. After the desodiation process, the lattice spacings narrowed to 1.835 and 1.84 Å, respectively, which correspond to the same lattice planes of NaVP with space group R$\bar{3}$c ^[^ ^42^ ^]^ (as shown in Figure 3b–d and Movie S1 in the Supporting Information). Figure S7 in the Supporting Information shows the intensity line profiles of (4$\bar{2}$6) and (1$\bar{3}$5) upon desodiation. This result indicates that the lattice contracted upon the extraction of Na^+^ ions. Figure 3e represents a particle of an intermediate phase, Na_2_V_2_(PO_4_)_3_ (Na_2_VP), with a different crystal structure from Na_3_VP and NaVP, which was observed outside the in situ observation area (inside the purple square in Figure 3a) after desodiating. This intermediate structure was consistent with a monoclinic structure with the P2_1_/c space group reported in a previous study.^[^ ^41^ ^]^ The values of unit cell volume per formula unit (*V*/*Z*) of Na_3_VP, Na_2_VP, and NaVP are 241.277,^[^ ^43^ ^]^ 227.159,^[^ ^41^ ^]^ and 220.105 Å^3^,^[^ ^41^ ^]^ respectively. The calculated volume changes from Na_3_VP to Na_2_VP and from Na_2_VP to NaVP were 5.9% and 3.1%, respectively, while the volume change in the phase transition between Na_3_VP and NaVP was 8.8%.

Upon Na_3_VP desodiating to Na_2_VP, the average V–O bond length in VO_6_ octahedron becomes longer, which contributes to the increase of the V–O octahedron volume. As Na_2_VP desodiates to NaVP, the V–O octahedron volume shrinks (as shown in Table S1 in the Supporting Information). It is suggested that although the value of *V*/*Z* gradually decreases with a decreasing number of Na^+^ ions, the larger VO_6_ octahedron volume in Na_2_VP causes a slight expansion in [V_2_(PO_4_)_3_]^3−^ framework. Therefore, the total volume changes from Na_3_VP to Na_2_VP and from Na_2_VP to NaVP (9.0%) is slightly larger than that from Na_3_VP to NaVP (8.8%). It is suggested that the gradual volume change, that is, from Na_3_VP to Na_2_VP and from Na_2_VP to NaVP, could reduce the lattice mismatch between Na_3_VP and NaVP, which will be further validated below. EDS mapping analysis of the desodiated sample showed that the Na signal of the cathode weakened after desodiation, indicating the extraction of Na^+^ ions (Figure 3f). Without an anode, the diffused Na^+^ ions through the NZSP accumulated around the Pt electrode (as shown in Figure 3f,h). The SAED images of the pristine and desodiated Pt electrodes showed the amorphousness of the electrodes (Figure S8, Supporting Information), indicating that the diffused Na^+^ ions did not alloy with Pt. The immobility of V indicates the structural integrity of the [V_2_(PO_4_)_3_]^3−^ framework (Figure 3g).

**Figure 4:**
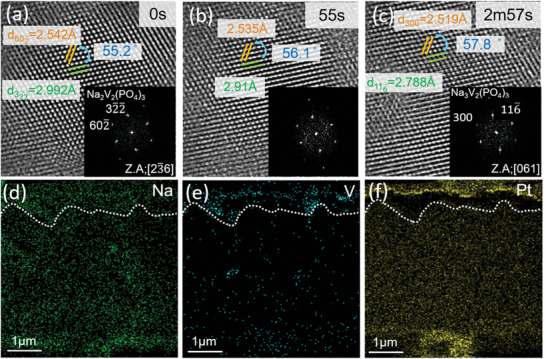
Sodiation of Na_2_VP. a–c) HRTEM images demonstrating the sodiation process of Na_2_VP. EDS images of d) Na, e) V, and f) Pt, respectively. The white dotted lines represent the interface between Na_3_VP and NZSP.

According to previous reports on layered sodium transition metal oxides, these materials undergo phase transitions owing to changes in the stacking sequence or the collapse of layered structures.^[^ ^44^ ^]^ As an example, Na*_x_* MnO_2_‐based oxides undergo detrimental phase transitions due to the impact of the Jahn–Teller effect of Mn^3+^.^[^ ^45^ ^]^ These results indicate a correlation between structural evolution (increasing disorder in and among the layers) and performance degradation. In Figure 3e, an intermediate Na_2_VP phase was observed, which also implies structural evolution during the desodiation process. However, the high stability and cycling ability of Na_3_VP has been demonstrated previously,^[^ ^46^ ^]^ which seems to contradict the studies mentioned above unless the occurring lattice distortions are reversible. Hence, an investigation of the phase transition between Na_2_VP and Na_3_VP is essential. **Figure** **4**a–c shows a series of HRTEM images of the phase transition from Na_2_VP to Na_3_VP, with the sample sodiated to 4 V. Initially, the lattice spacings were 2.542 and 2.992 Å, corresponding to the (60$\bar{2}$) and (3$\bar{2}\bar{2}$) lattice planes of the Na_2_VP phase. During sodiation, the dihedral angle gradually changed from 55.2° to 57.8°. The lattice spacings were 2.519 and 2.788 Å after sodiation for the (300) and (11$\bar{6}$) lattice planes of the Na_3_VP phase, respectively (the sodiation process is shown in Movie S2 in the Supporting Information). Figure S9 (Supporting Information) shows the intensity line profiles of (60$\bar{2}$) ((300)) and (3$\bar{2}\bar{2}$) ((11$\bar{6}$)) upon sodiation. The EDS mapping of the sodiated sample showed that the Na signal became stronger (Figure 4d), whereas the Na signal around the Pt electrode was weakened (as shown in Figure 4d,f). This indicates that the Na^+^ ions diffused from NZSP toward cathode during sodiation. The distribution of V remained unchanged after sodiation (Figure 4e), further confirming the structural stability of Na_2_VP/Na_3_VP.

The solid–solid contact between the electrode and electrolyte has been a crucial issue for all‐solid‐state batteries.^[^ ^47^ ^]^ The interfacial stress induced by the volume changes of active materials during sodiation/desodiation ruptures the interface between the electrolyte and electrode. As the cracks at the contacted interface form, the cross‐section of the interface decreases, increasing the impedance of the interface. The increasing impedance causes more Joule heating, leading to a vicious cycle. In Figure S10 in the Supporting Information, there was no visible crack formation at the interface between Na_3_VP and NZSP after one cycle. To verify the continuity of the interface, the resistances of the sample were measured under a constant bias of 4 V for 15 min. The resistance showed no obvious increase during desodiation (Figure S11a, Supporting Information), and sodiation (Figure S11b, Supporting Information), suggesting that the contact between Na_3_VP and NZSP remained intact after cycling. Notably, the resistances decreased slightly and then increased in both the desodiated and sodiated curves (as shown in Figure S11c,d, respectively, Supporting Information). To clarify the effect of intermediate Na_2_VP phase on the interface, an in‐depth experiment has been conducted. The sample was sodiated/desodiated to 4 V for three cycles. As the magnification becomes higher, the electron current density (dose rate) becomes larger, causing the structural degradation of cathode material.^[^ ^48^ ^]^ To avoid this situation, the in situ area with constant electron beam irradiation (to avoid the formation of Na_2_VP) was observed under relative low magnification during applying bias. After reaching 4 V, the interface was further observed with higher magnification. After three cycles, the interface outside the in situ area (with Na_2_VP) remained continue (Figure S12a, Supporting Information) while the voids started to form along the interface within in situ area (without Na_2_VP) (Figure S12b, Supporting Information). It is suggested that the larger volume change between Na_3_VP and NaVP causes severer structural collapse than that from Na_3_VP to Na_2_VP and from Na_2_VP to NaVP. The lattice mismatch at the boundary of the intact and degraded phases cause the lattice strain that eventually leads to void formation during repeated charge/discharge.

**Figure 5:**
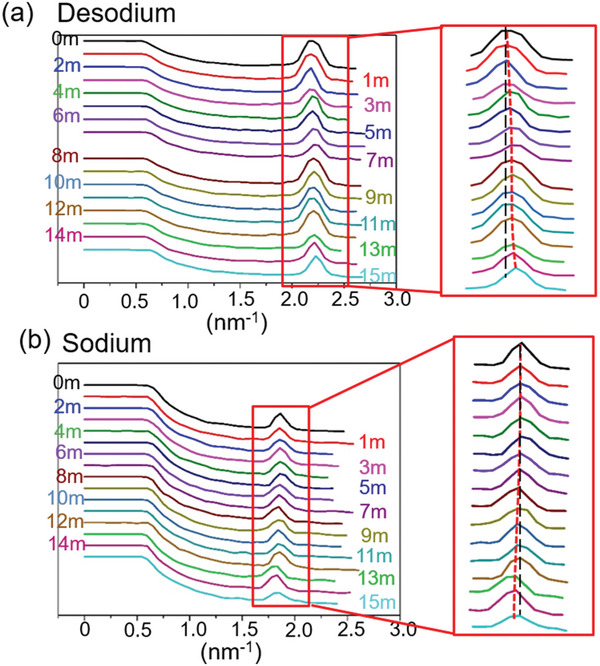
Characterization of the sodiation/desodiation reaction of the Na_3_VP cathode. The distances between the transmitted beams and the diffraction spots during a) desodiation and b) sodiation. The black dashed lines represent perpendicular lines.

To further validate the sodiation/desodiation reactions of Na_3_VP, the SAED patterns of the Na_3_VP were analyzed at a constant voltage of 4 V for 15 min. Figure S13 in the Supporting Information shows a series of SAED images of the Na_3_VP during desodiation. The spectrum in **Figure** **5**a in the Supporting Information depicts the intensity from the transmitted beams to the diffraction spots (marked by the green arrows in Figure S13 in the Supporting Information). The bright diffraction spot has a high intensity because the intensity is determined by the brightness contrast of the SAED image. Therefore, the diffraction spot in Figure S13 in the Supporting Information forms a peak in the spectrum of Figure 5a in the Supporting Information. The peak shifted slightly toward the right during the desodiation process, indicating an increase in the distance between the transmitted beam and the diffraction spot. In the reciprocal space, the distance from a diffraction spot to the transmitted beam is proportional to the reciprocal of the planar space. Therefore, it is suggested that the lattice spacing of Na_3_VP decreased when the Na^+^ ions were extracted. This was consistent with the in situ TEM results. Na_3_VP was then sodiated for 15 min, and the corresponding SAED images are shown in Figure S14 in the Supporting Information. The left shift of the peak (Figure 5b) shows a decrease in the distance between the transmitted beam and the diffraction spot in Figure S14 in the Supporting Information. In other words, the lattice spacing of Na_3_VP expanded during the insertion of Na^+^ ions.

**Figure 6:**
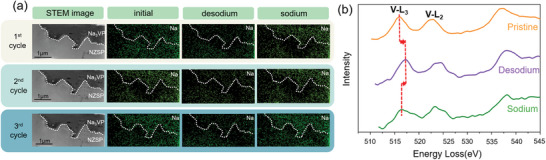
EDS mapping and EELS spectra of Na_3_VP. a) The STEM image of the sample and their corresponding EDS mapping of Na during three cycles. The white dotted lines represent the interface between Na_3_VP and NZSP. b) EELS spectra of the V L‐edge of the pristine, desodiated, and sodiated states of Na_3_VP.

In addition to the reversibility of the sodiation/desodiation process as confirmed via the in situ TEM and SAED patterns, rechargeability is also an important quality for a secondary battery. For the TEM sample fabricated by FIB, the sample has a lamellar shape and a thickness of ≈100 nm. The lamellar shape has a much smaller cross‐sectional area compared with the bulk Na‐ASSB, resulting in a higher current density. Moreover, contacts of Pt wires and the sample (as shown in Figure S15a in the Supporting Information), where the current direction and current density change, suffer from the current crowding effect. Therefore, thermal stresses could affect the in situ TEM sample significantly, which may deteriorate its rechargeability. To minimize the thermal impact on the samples, Pt pads with a side length of 1.5 µm were deposited on the contacts via FIB (Figure S15b–d, Supporting Information). The Pt pads were expected to increase the contact area between the Pt wires and the sample and reduce the change in the current direction. **Figure** **6**a shows the STEM images and Na mapping of the sample from the first to the third cycle. The changes in the intensity of the Na signal in Na_3_VP verified the release/uptake of Na^+^ ions during the desodiation/sodiation process, while the unchanged V distribution (Figure S16, Supporting Information) and the corresponding HRTEM images of Na_3_VP after cycling (from the first to the third cycle) shown in Figure S17 in the Supporting Information validated the stability of the NASICON structure. After cycling, the interface between Na_3_VP and NSZP still exhibited excellent contact. It is suggested that the optimization of the in situ device mitigated the thermal effect on the sample, improving its rechargeability. EELS was utilized to investigate the valence states of V. Figure 6b shows the V‐L_2,3_ spectra of pristine, desodiated, and sodiated Na_3_VP. The L‐edge shifted toward higher energies due to Na^+^ ion extraction. The valence state of V changed from +3 to +4, which corresponds to the desodiation from Na_3_VP to NaVP. The L‐edge shifted toward lower energies when the Na^+^ ions were inserted, suggesting the recurrence of Na_3_VP.^[^ ^49^ ^]^

**Figure 7:**
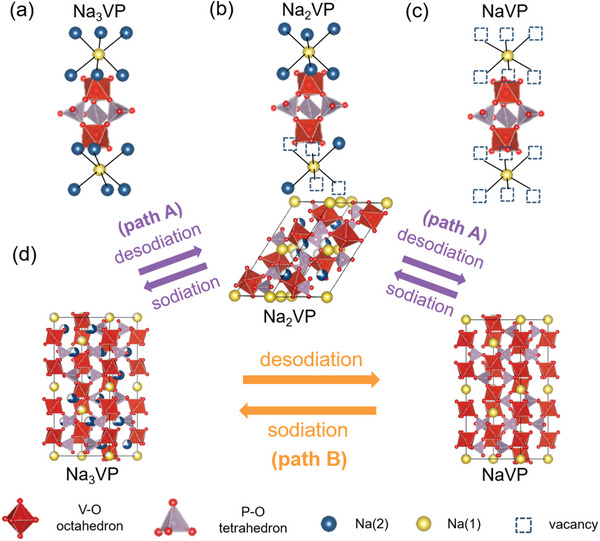
Reaction mechanism of Na_3_VP cathode. The lantern unit and the occupancy of Na^+^ ions in a) Na_3_VP, b) Na_2_VP, and c) NaVP. d) Schematic diagram showing the evolution of structure during the sodiation/desodiation of Na_3_VP.

For a better understanding of the mechanism of the phase transition in the Na_3_VP–NaVP system, an in‐depth discussion of the structural transformation is necessary. Each Na_3_VP structural unit can accommodate three Na atoms, where one is located at the Na(1) site and the other two are located at the Na(2) sites. The Na(2) ions are considered to be the main participants in the transportation of Na^+^ ions because of their longer Na–O distance than that of the Na(1) site. **Figure** **7**a–c shows the structural evolution after the Na^+^ ions were extracted. The lantern unit consists of two VO_6_ octahedra sharing corners with three PO_4_ tetrahedra. The yellow and blue spheres represent Na^+^ ions at the Na(1) and Na(2) sites, respectively. In the rhombohedral Na_3_VP, both Na(1) and Na(2) sites are partially occupied (Figure 7a). During the desodiation process, Na^+^ ions are extracted from the Na(2) sites, resulting in less occupancy of this position. The extraction of the Na(2) ions reduces the rhombohedral symmetry due to a distortion of the lattice, transforming the rhombohedral structure into a monoclinic structure (Figure 7b). Once all Na^+^ ions are extracted from the Na(2) sites, the monoclinic structure then transforms back to the rhombohedral phase (Figure 7c), owing to the symmetry of the vacant Na(2) sites. Furthermore, DFT computations were employed to determine the formation energies of the NaVP crystals along the Na_3_VP and Na_2_VP pathways. Among the different structures, Na_3_VP has the highest formation energy (−2.229 eV per atom), while Na_2_VP and NaVP manifest lower formation energies of −2.264 and −2.280 eV per atom, respectively. This indicates that the Na_3_VP cathode prefers to transform to the Na_2_VP or NaVP structures. Moreover, the desodiation energy from Na_3_VP to Na_2_VP is much lower than that from Na_3_VP to NaVP, demonstrating that the Na_3_VP structure is more likely to transform to Na_2_VP structure during the desodiation process (path A in Figure S18 in the Supporting Information) rather than the NaVP structure (path B). Figure S19 in the Supporting Information shows the diffusion barriers for the Na^+^ ion migration in the Na_2_VP and Na_3_VP structures. The energy barrier for Na^+^ ion diffusion in the Na_2_VP crystal is 0.138 eV, which is smaller than that in Na_3_VP (0.298 eV). For NaVP, where all Na ions only occupy Na(1) sites, the extraction of Na ions may affect the structural integrity. Therefore, Na migration between Na(1) sites at NaVP is hindered by a higher migration barrier than Na_3_VP.^[^ ^50^ ^]^ This suggests that the Na^+^ ion migrates more rapidly in the Na_2_VP structure compared to Na_3_VP and NaVP, thereby decreasing the resistance of the sample (Figures S11c,d, Supporting Information).

Based on the results of the in situ TEM, Na_3_VP can transform to NaVP with or without the Na_2_VP phase. This behavior could be attributed to electron beam irradiation. As mentioned above, high magnification of TEM could cause the degradation of cathode.^[^ ^48^ ^]^ The state of the aperture affects not only the number of incident electrons on the sample, but it also affects the area of irradiation. Therefore, the electron current density (dose rate) remain the same in spite of the state of the aperture. Although the electron irradiation of TEM may cause irreversible damage to the sample,^[^ ^51^ ^]^ it can provide a feasible stimulus that mimics the effect of electrochemical cycling.^[^ ^52^ ^]^ Huang et al. reported that electron beams could reclaim Li metal from the degradation product and serve as a local Li source, which triggers the lithiation of NiFe_2_O_4_/carbon nanotubes.^[^ ^53^ ^]^ LiNi_0.4_Mn_0.4_Co_0.18_Ti_0.02_O_2_ particles, repeated electron beam irradiation induced a phase transition from an R$\bar{3}$m layered structure to an Fm$\bar{3}$m rock‐salt structure, which is attributed to the stoichiometric lithium and oxygen removal from R$\bar{3}$m 3a and 6c sites, respectively.^[^ ^54^ ^]^ This implies that the electron beam can be used as a high‐energy particle flow to change the microstructure of sensitive materials. In Figure 3b–d, the Na_3_VP particle was exposed to a high‐energy electron beam throughout the entire desodiation process. Na^+^ ions could be easily extracted from Na_3_VP owing to the interaction between electrons and atoms. Therefore, the Na_3_VP phase could transform to the NaVP phase directly without the formation of the intermediary Na_2_VP phase (path B in Figure 7d). Meanwhile, the Na_3_VP outside the in situ observation region, where the effect of electron beam irradiation was insignificant, transformed to Na_2_VP, as shown in Figure 3e, thus lowering the formation energy of NaVP (path A in Figure 7d).

## 3 Conclusions

In conclusion, a Na_3_VP/NZSP cell with excellent solid–solid contact was fabricated. The charging/discharging process of Na_3_VP was investigated using operando XRD. The structural evolution of Na_3_VP was demonstrated with in situ TEM. The lattice spacing decreased during Na^+^ ion extraction, which corresponds to the transformation of Na_3_VP to NaVP. Outside the in situ area, the intermediary Na_2_VP phase with the space group P2_1_/c was observed at atomic scale. Na_2_VP could reduce the lattice mismatch between Na_3_VP and NaVP, thereby preventing structural collapse. Therefore, the interface between Na_3_VP and NZSP remained intact with the formation of Na_2_VP phase. The mechanism of the phase transition from Na_2_VP to Na_3_VP was revealed by analyzing the structural evolution during sodiation. In addition to the lattice spacing, the dihedral angle was also altered when the Na^+^ ions were inserted. The formation of the intermediate Na_2_VP phase can be attributed to the asymmetry of Na(2) sites in the rhombohedral structure. The Na_2_VP phase lowers the formation energy of NaVP and possesses lower energy barrier for Na^+^ diffusion. This study allows a better understand of the behavior of fast‐rate electrode materials that undergoes phase transition, providing insight into the future design of Na_3_VP.

## 4 Experimental Section

### Na_3_VP and NZSP Preparation

NZSP powder was synthesized on a laboratory scale (10 g^−1^ kg per batch) using a solution‐assisted solid‐state reaction method (SA‐SSR).^[^ ^6^, ^55^ ^]^ NaNO_3_ (VWR), ZrO(NO_3_)_2_ (Aldrich), Si(OCH_2_CH_3_)_4_ (Merck), and NH_4_H_2_PO_4_ (Merck) were used as starting materials. All the chemicals applied in this study were analytically pure. Stoichiometric amounts of NaNO_3_ and ZrO(NO_3_)_2_ were dissolved in deionized water. A stoichiometric amount of Si(OCH_2_CH_3_)_4_ was also added to the solution while stirring. When Si(OCH_2_CH_3_)_4_ was hydrolyzed, the stoichiometric amount of NH_4_H_2_PO_4_ was added to the system during stirring. The homogeneous aqueous solution immediately showed formation of complex zirconium oxyphosphate compounds. The whole mixture was dried at 85 °C. The dried powder was then calcined at 800 °C for 3 h. After calcination, a white powder was obtained. The calcined powder was then milled in ethanol with zirconia balls on a milling bench for 48 h, and dried at 70 °C for 12 h.

The NZSP powder was put into a cylindrical pressing mold (13 mm in diameter) and pressed with a uniaxial pressure of about 100 MPa at room temperature. The pressed pellets were sintered between 1260 and 1300 °C for 5 h. The obtained pellets had a diameter of about 10 mm, and thickness of 1–2 mm. The relative density of the sintered pellets was >95%.

The Na_3_VP precursor solution was prepared by mixing ethanolamine, de‐ionized H_2_O, NaH_2_PO_4_, and NH_4_VO_3_ (weight ratios of 1:2:0.71:0.46). A cotton swab was dipped into the Na_3_VP precursor solution and then swept over the surface of the sintered NZSP pellet, leaving a thin and homogenous Na_3_VP precursor solution layer on the NZSP pellet. The pellets were then heated to 740 °C in Ar‐2% H_2_ for 4 h to form the Na_3_VP phase on the NZSP surface.

### Operando XRD Measurement

For the operando XRD analysis, Au was then sputtered on the positive electrodes to serve as current collector. The cell was assembled inside Ar‐filled glove box, subjected into X‐ray examination during charging/discharging at a rate of 0.4 C. Bruker D2 Phaser and BioLogic SP‐150 Potentiostat are used as X‐ray diffraction instrument and a modulo‐battery‐mode electrochemical tester, respectively.

### In Situ TEM Observation

An in situ TEM sample of Na_3_VP/NZSP was prepared using a high‐resolution dual‐beam focused ion beam (FIB) system (FEI Versa 3D). Because of the unevenness of the Na_3_VP/NZSP sample (Figure S20a, Supporting Information), a Pt protection layer was deposited first to smoothen the surface using the FIB technique. The second Pt layer was then deposited onto the first Pt layer to prevent damage from the Ga ions (Figure S20b, Supporting Information). The trenches were milled on both sides of the lamella, as shown in Figure S18c in the Supporting Information. A low‐kV cleaning process was used at the end of the FIB process (Figure S20d, Supporting Information) to reduce the influence of Ga ions. The sample was then transferred by a glass tip onto the in situ TEM chip, and Pt wires were deposited by the FIB system to connect the electrodes of the chip to those of the TEM sample (Figure S20e, Supporting Information). An enlarged scanning electron microscopy (SEM) image of the TEM sample is shown in Figure S20f in the Supporting Information. Subsequently, the chip was mounted onto an in situ TEM holder (Protochips Audro300). In situ TEM movies and images were recorded using a JEOL F200 system equipped with EDS using OneView CCD.

### Characterization of Pristine/Sodiated/Desodiated Na_3_VP

X‐ray diffraction (XRD) pattern of the pristine sample was measured using a Brucker D2 Phaser with Cu Kα radiation. After the samples were sodiated/desodiated to 4 V, the valence states of V in Na_3_VP were characterized using EELS.

### Density Functional Theory Calculation

First‐principles calculations were conducted by using the Vienna ab initio simulation package (VASP)^[^ ^56^, ^57^ ^]^ with the projector augmented wave (PAW)^[^ ^58^ ^]^ method based on the density functional theory (DFT). The Perdew–Burke–Ernzerhof (PBE)^[^ ^59^ ^]^ functional within the generalized‐gradient approximation (GGA)^[^ ^60^ ^]^ was used to describe the exchange‐correlation interaction for the structure optimization. The cut‐off energy was set to 600 eV for all the calculations. The first Brillouin zone was sampled with 3 × 3 × 1, 2 × 3 × 3, and 3 × 3 × 1 *k*‐points grids for Na_3_VP, Na_2_VP, and NaVP crystals, respectively. The energy convergence criterion was set as 1.0 × 10^−5^ for all the calculations. The optimized lattice parameters (*a* = *b* = 8.832 Å, *c* = 22.094 Å for Na_3_VP; *a* = 15.432 Å, *b* = 8.718 Å, *c* = 8.851 Å for Na_2_VP; *a* = *b* = 8.496 Å, *c* = 21.656 Å for NaVP) are good consistent with the experimental values.^[^ ^35^, ^41^ ^]^

The formation energy (*E*_f_) of the NaVP crystal is expressed by Equation (2)

(2) $$E_{f}=\left(E_{{\mathrm{Na}}_{x}V_{2}{\left({\mathrm{PO}}_{4}\right)}_{3}}-\sum_{i}n_{i}\mu_{i}\right)/N$$

where $E_{{\mathrm{Na}}_{x+y}V_{2}{\left({\mathrm{PO}}_{4}\right)}_{3}}$ and *N* are the total energy and the total number of atoms of the NaVP crystals per formula units (*x* = 1, 2, 3); *n_i_* and *µ_i_* are the number of atoms and the chemical potential of the species *i*, respectively. The chemical potentials of Na, V, and P were conducted using the total energy per atom in the bulk system, and that of O was obtained using O_2_ molecule.

The desodiation energy (*E*_D_) of the NaVP crystal during the desodiation process is expressed by Equation (3)

(3) $$E_{D}=E_{{\mathrm{Na}}_{x}V_{2}{\left({\mathrm{PO}}_{4}\right)}_{3}}+y\mu_{\mathrm{Na}}-E_{{\mathrm{Na}}_{x+y}V_{2}{\left({\mathrm{PO}}_{4}\right)}_{3}}$$

where $E_{{\mathrm{Na}}_{x+y}V_{2}{\left({\mathrm{PO}}_{4}\right)}_{3}}$ is the total energy of the Na*_x_*_+_ *_y_* VP crystals per formula units (*x* = 1, 2, 3; *y* = 1, 2)

The transition‐state (TS) was determined using the climbing image nudged elastic band (CI‐NEB) calculation through the VTST code^[^ ^61^, ^62^ ^]^ and the diffusion barriers related to the energy difference between the initial‐state (IS) and the TS. All atoms were allowed to relax until the force components were less than 0.05 eV Å^−1^.

## Conflict of Interest

The authors declare no conflict of interest.

## Supporting information

Supporting Information

Supplemental Movie 1

Supplemental Movie 2

## Data Availability

The data that support the findings of this study are available from the corresponding author upon reasonable request.
