# Supplementary material for: In Situ Atomic‐Scale Investigation of Structural Evolution During Sodiation/Desodiation Processes in Na3V2(PO4)3‐Based All‐Solid‐State Sodium Batteries
Source: Adv Sci (Weinh). 2023 Sep 6;10(32):2301490. doi: 10.1002/advs.202301490 (PMC10646283; doi:10.1002/advs.202301490)
Supplement: Supplementary file 1 — Supporting Information [file ADVS-10-2301490-s003.pdf]

ADVANCED SCIENCE
Open Access

Supporting Information

for *Adv. Sci.*, DOI 10.1002/advs.202301490

In Situ Atomic-Scale Investigation of Structural Evolution During Sodiation/Desodiation Processes in $Na_3V_2(PO_4)_3$-Based All-Solid-State Sodium Batteries

*Fang-Chun Shen, Qianli Ma, Frank Tietz, Jui-Cheng Kao, Chi-Ting Huang, Rahmandhika Firdauzha Hary Hernandha, Chun-Wei Huang, Yu-Chieh Lo, Jeng-Kuei Chang and Wen-Wei Wu**

Supporting Information

In Situ **Atomic-Scale Investigation of Structural Evolution during Sodiation/Desodiation Process in $Na_3V_2(PO_4)_3$-Based All-Solid-State Sodium Batteries**

*Fang-Chun Shen, Qianli Ma, Frank Tietz, Jui-Cheng Kao, Chi-Ting Huang, Rahmandhika Firdauzha Hary Hernandha, Chun-Wei Huang, Yu-Chieh Lo, Jeng-Kuei Chang, Wen-Wei Wu**

List of Contents

Figure S1|Schematic diagram showing the all-solid-state Na_3VP/NZSP samples.

Figure S2|XRD pattern of the Na_3VP/NZSP sample.

Figure S3|Schematic diagram showing the structures of Na_3VP and NZSP.

Figure S4|TEM images of the pristine interface between Na_3VP and NZSP..

Figure S5|Cycling performances of Na_3VP-NZSP-Na cells operating at 25 °C.

Figure S6|I*n situ* XRD patterns of Na_3VP during first cycle with a voltage window of 2.7 – 4.0 V vs. Na^+/Na at the C-rate of 0.4 C.

Figure S7|The intensity line profiles of Na_3VP during desodiation.

Figure S8|SAED images of the Pt electrode.

Figure S9|The intensity line profiles of Na_2VP during sodiation.

Figure S10|TEM images of the interface between Na_3VP and NZSP after a cycle.

Figure S11|Resistance measurements of the sample at 4 V for 15 minutes.

Figure S12|TEM images of the interface between Na_3VP and NZSP after three cycles.

Figure S13|SAED images of Na_3VP and the distances between two diffraction spots during desodiation.

Figure S14|SAED images of Na_2VP and the distances between two diffraction spots during sodiation.

Figure S15|SEM images of the TEM sample with Pt wires deposited with the FIB system.

Figure S16|STEM images of the sample and EDS mapping of the V signals.

Figure S17|HRTEM images of cathode.

Figure S18|Schematic diagram illustrating the evolution of the Na_3VP crystal structure during the desodiation process, along with the corresponding formation energy (black digits) and the desodiation energy (blue digits).

Figure S19|Migration energy profiles of the Na^+ ion in the (a) Na_2VP and (b) Na_3VP crystals, as well as the corresponding initial-state (IS), transition-state (TS), and final-state (FS) structures.

Figure S20|SEM images showing the preparation of the TEM sample and the deposition of electrodes with the FIB system.

Table S1. Unit cell parameters, average V–O distances and V-O octahedron volumes for the compositions NaxVP, (x = 1, 2, and 3)

Movie S1|Desodiation process of Na_3VP at the atomic scale.

Movie S2|Sodiation process of Na_2VP at the atomic scale.

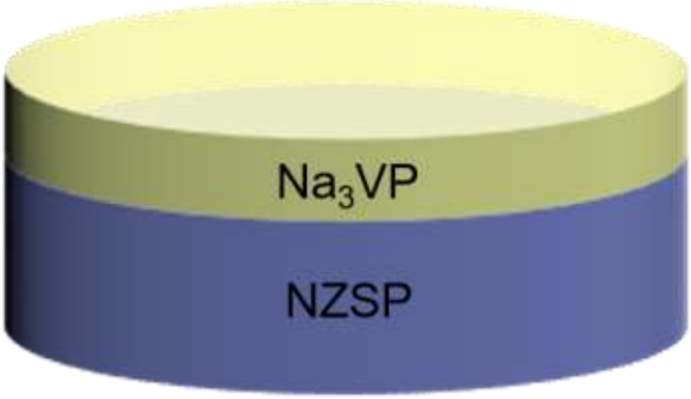

Figure S1. Schematic diagram showing the all-solid-state Na_3VP/NZSP samples.

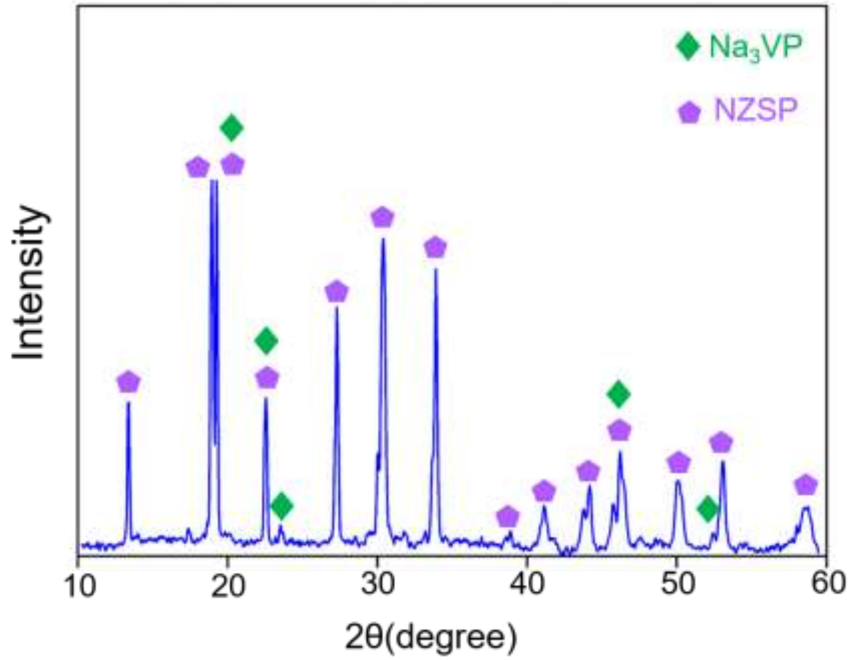

Figure S2. XRD pattern of the Na_3VP/NZSP sample.

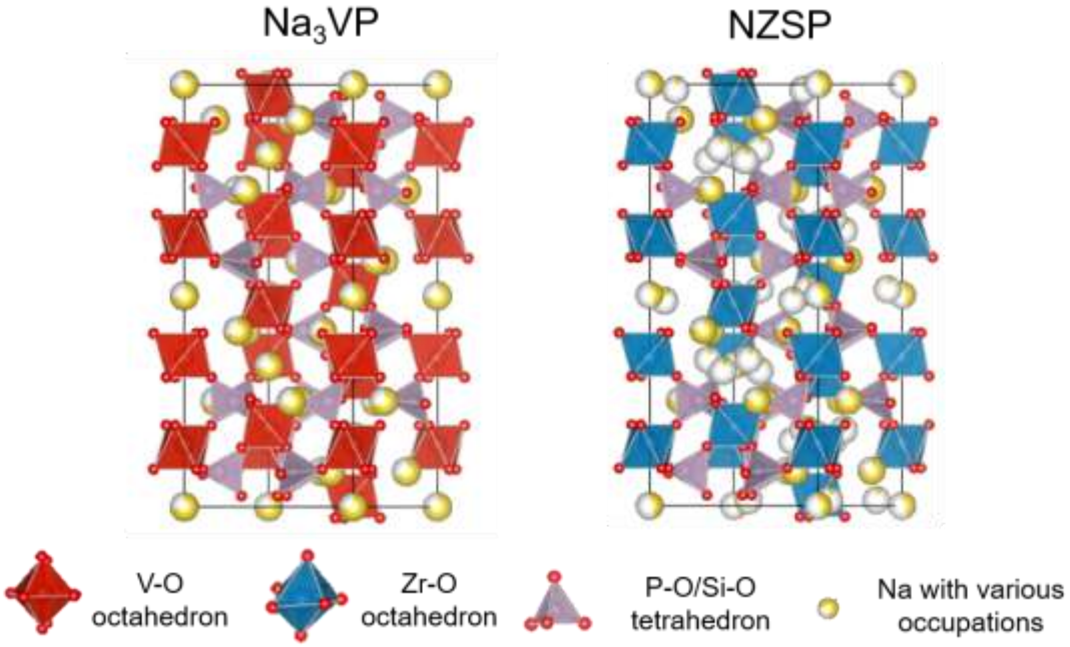

Figure S3. Schematic diagram showing the structures of Na_3VP and NZSP.

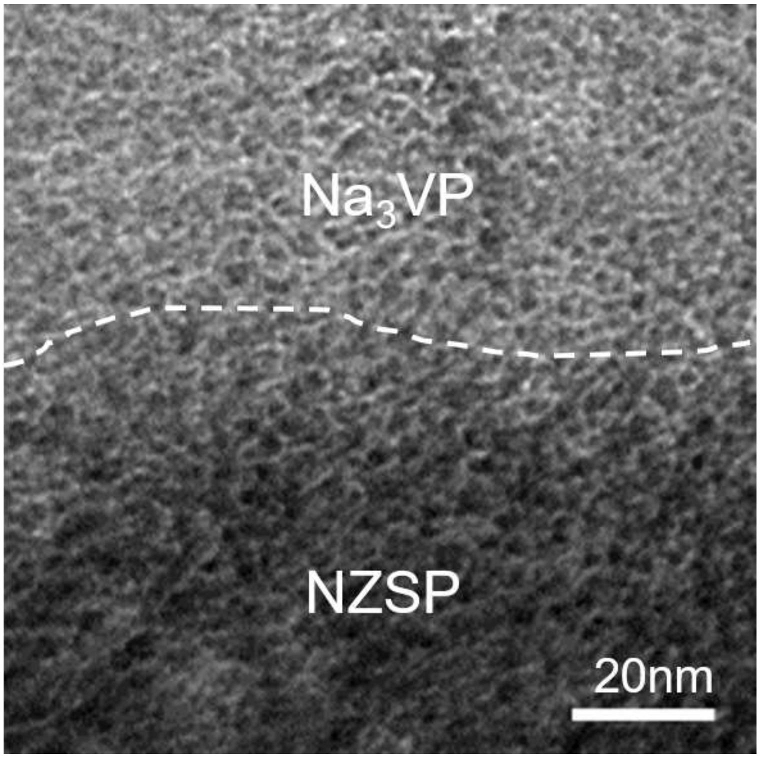

Figure S4. TEM images of the pristine interface between Na_3VP and NZSP.

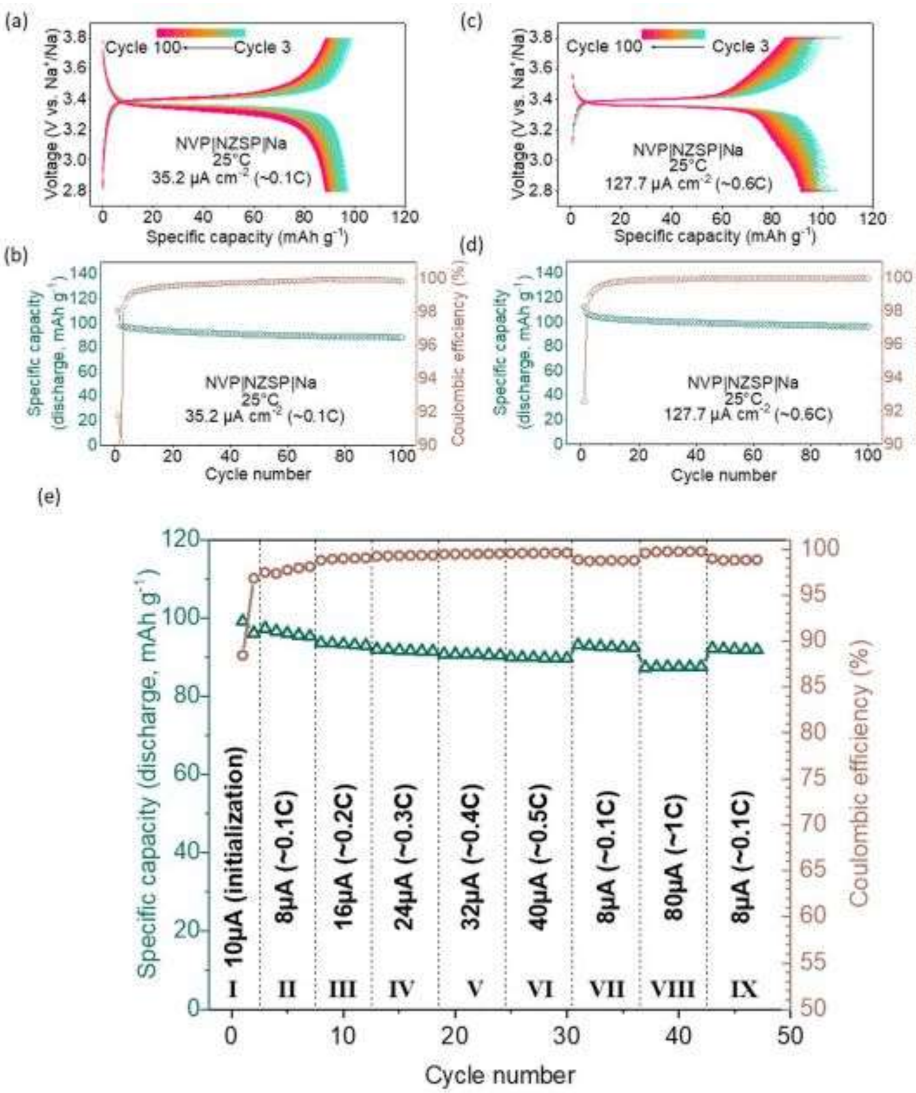

Figure S5. Cycling performances of Na_3VP-NZSP-Na cells operating at 25 °C. (a–b) performance of a cell operating with 35.2 μA cm^{-2}: (a) Charge and discharge curves for stable cycles (no. 3–100); (b) Discharge capacity and Coulombic efficiency for each cycle. (c–d) Performance of a cell with a current density of 127.7 μA cm^{-2}: (c) Charge and discharge curves for stable cycles (no. 3–100); (d) Discharge capacity and Coulombic efficiency for each cycle. (e) Performance of a cell operating with different current densities: corresponding discharge capacity and Coulombic efficiency for each cycle. (Nano Energy 2019, 65, 104040)

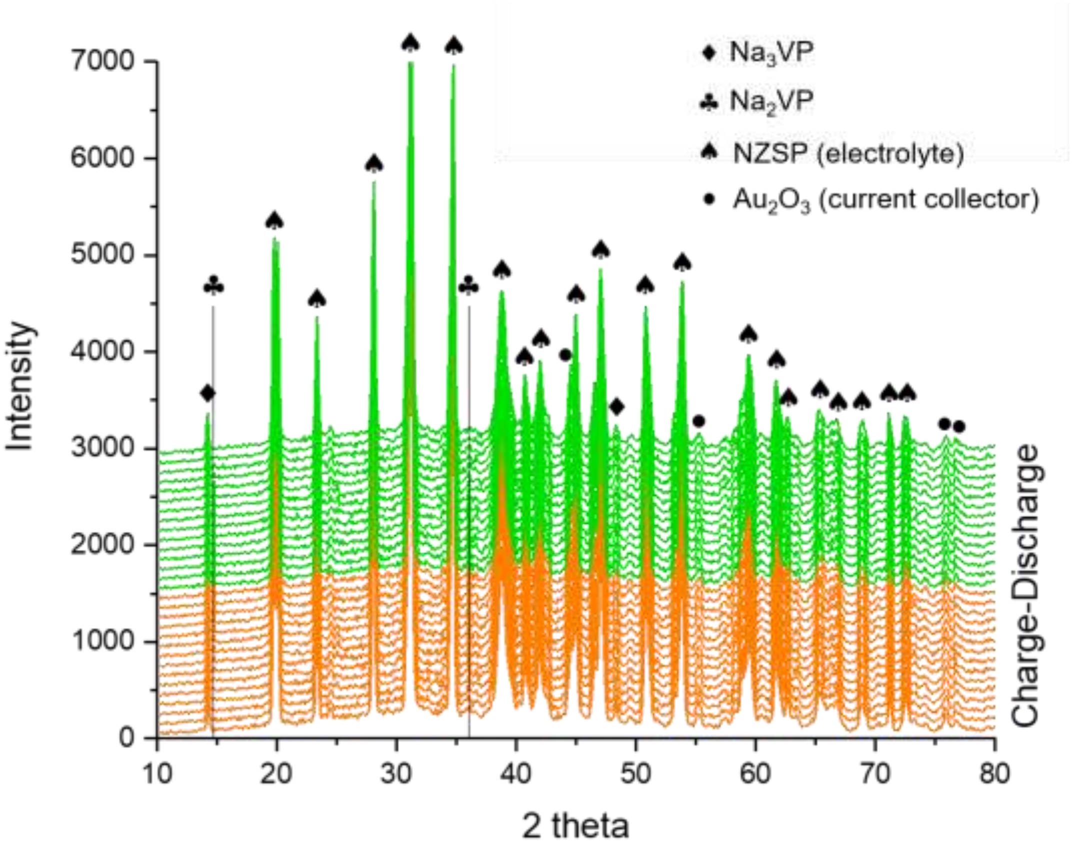

Figure S6. I*n situ* XRD patterns of Na_3VP during first cycle with a voltage window of 2.7 – 4.0 V vs. Na^+/Na at the C-rate of 0.4 C.

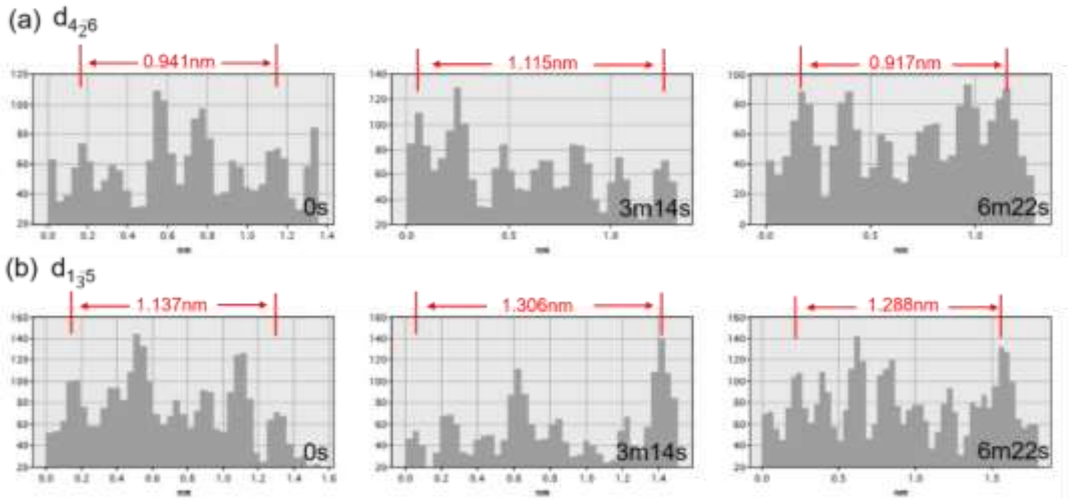

Figure S7. The intensity line profiles of Na_3VP during desodiation. (a) The evolution of $d_{4\bar{2}6}$. (b) The evolution of $d_{1\bar{3}5}$.

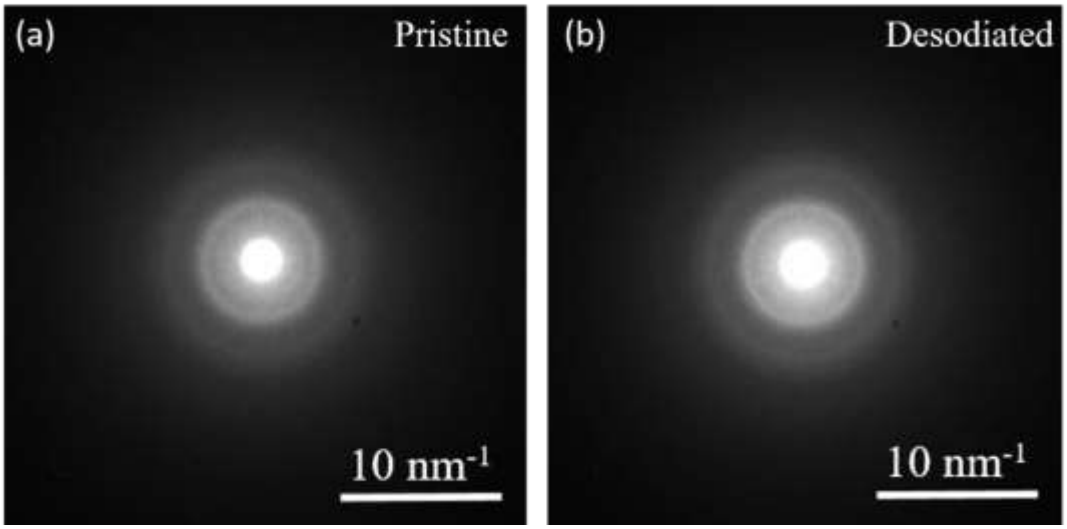

Figure S8. SAED images of the Pt electrode. (a) Pristine Pt electrode. (b) Pt electrode after the sample was desodiated.

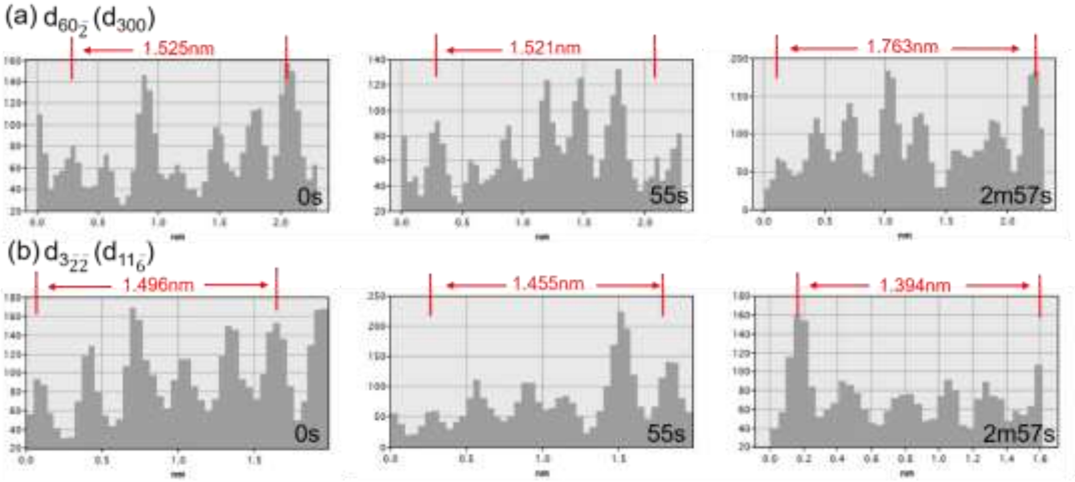

Figure S9. The intensity line profiles of Na_2VP during sodiation. (a) The evolution of $d_{60\bar{2}}$ (d_{300}). (b) The evolution of $d_{3\bar{2}\bar{2}}$ ($d_{11\bar{6}}$).

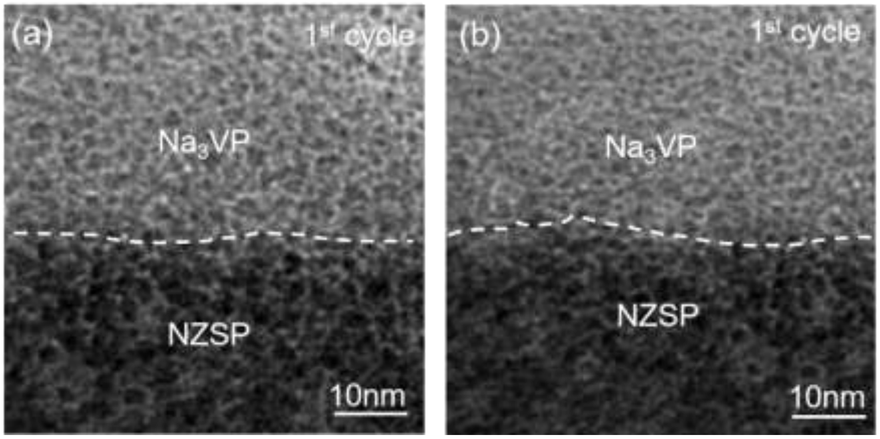

Figure S10. TEM images of the interface between Na_3VP and NZSP after a cycle. (a) Interface of the sample outside the *in situ* area (with Na_2VP phase). (b) Interface of the sample within the *in situ* area (without Na_2VP phase). The white dashed lines indicate the interfaces between Na_3VP and NZSP.

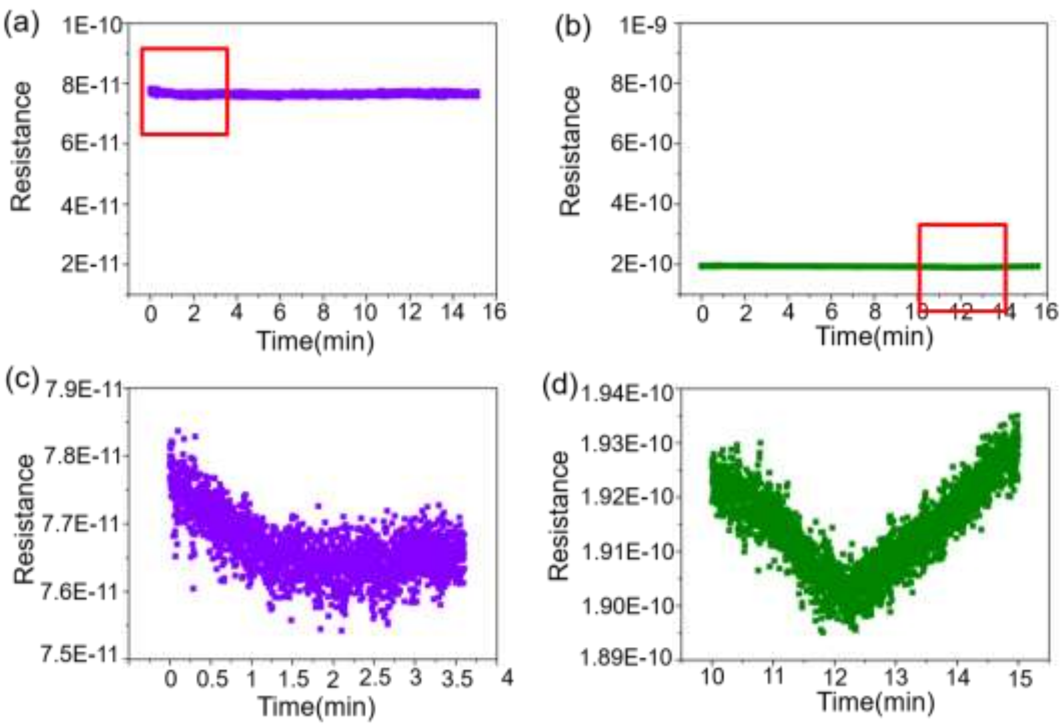

Figure S11. Resistance measurements of the sample at 4 V for 15 minutes. Resistance of the sample during (a) desodiation and (b) sodiation. Resistance of the sample from 0 to 4 minutes during (c) desodiation and from 10 to 15 minutes during (d) sodiation.

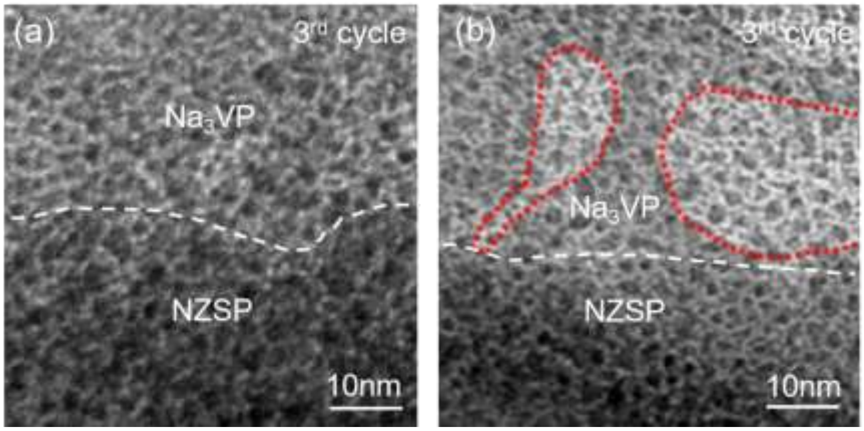

Figure S12. TEM images of the interface between Na_3VP and NZSP after three cycles. (a) Interface of the sample outside the *in situ* area (with Na_2VP phase). (b) Interface of the sample within the *in situ* area (without Na_2VP phase). The white dashed lines indicate the interfaces between Na_3VP and NZSP.

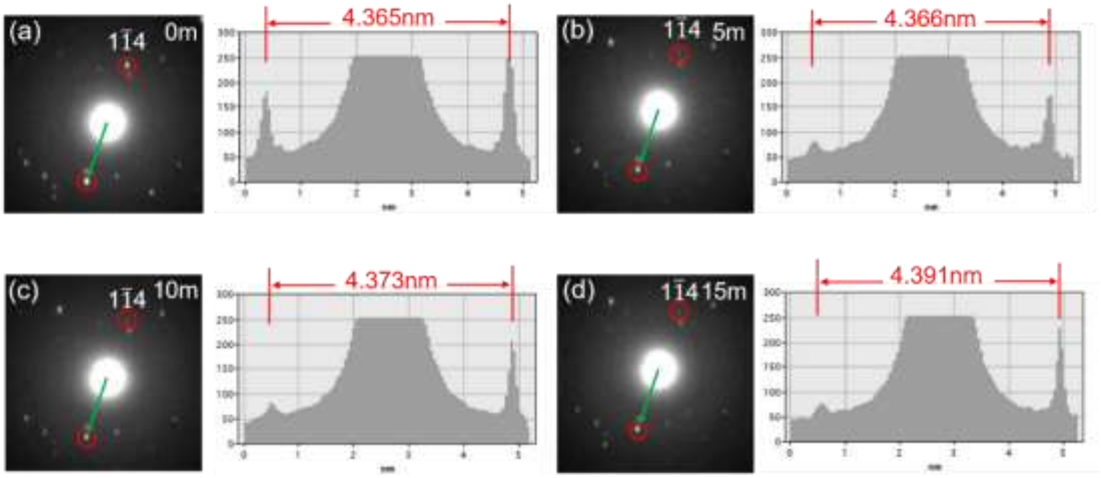

Figure S13. SAED images of Na_3VP and the distances between two diffraction spots during desodiation. (a–d) SAED images of Na_3VP during the desodiation process at 4 V for 15 minutes. The distances between two diffraction spots circled in red lines are shown on the right of the corresponding SAED images.

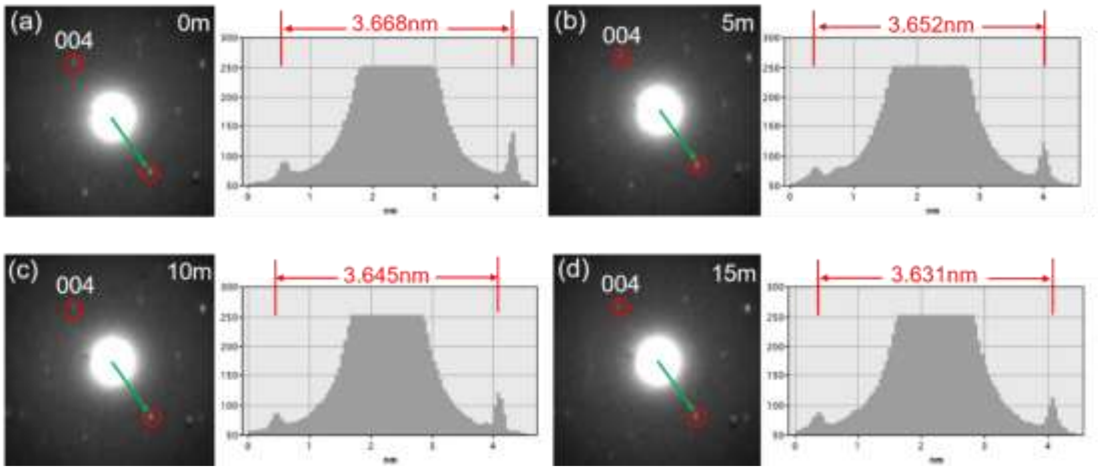

Figure S14. SAED images of Na_3VP and the distances between two diffraction spots during sodiation. (a–d) SAED images of Na_3VP during the sodiation process at 4 V for 15 minutes. The distances between two diffraction spots circled in red lines are shown on the right of the corresponding SAED images.

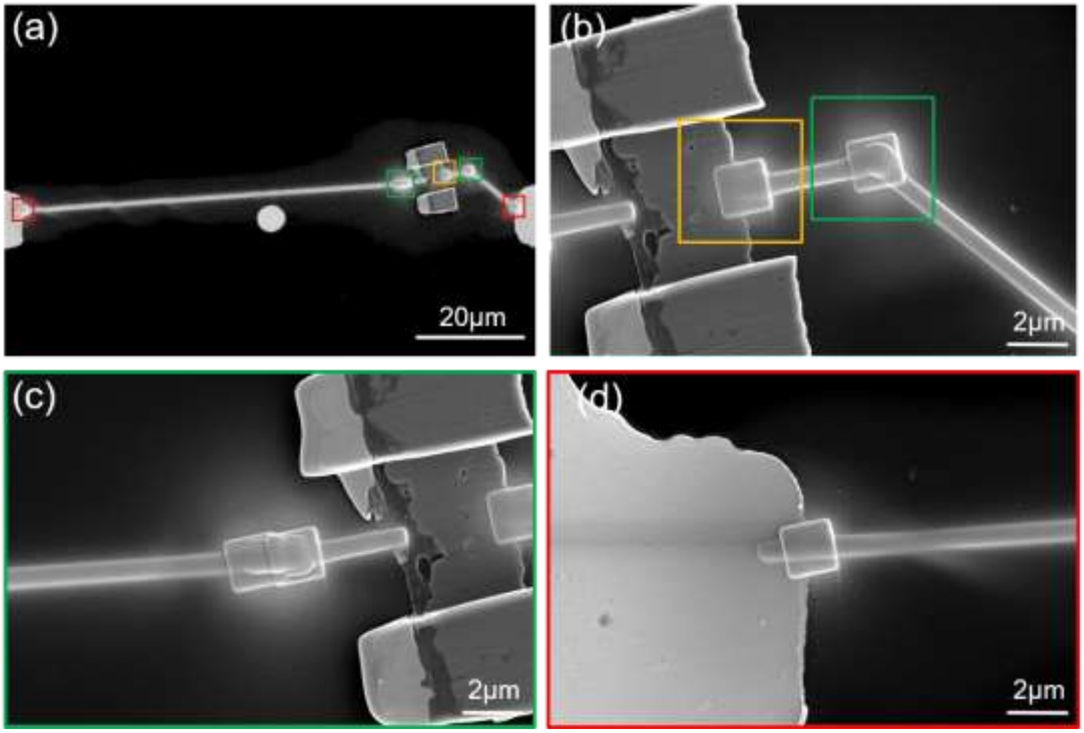

Figure S15. SEM images of the TEM sample with Pt wires deposited with the FIB system. (a) The full image of the TEM sample and Pt wires. (b–d) Enlarged images showing the contacts of the sample and Pt wires.

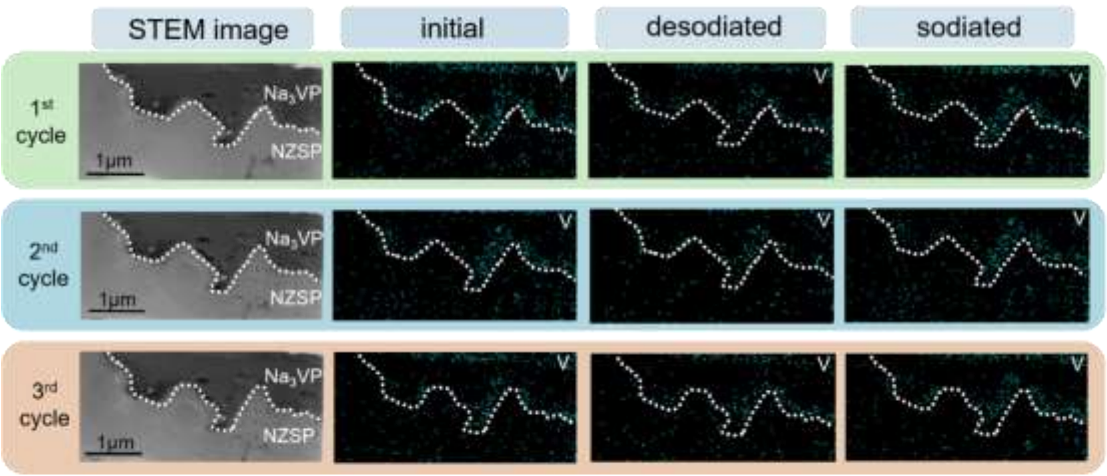

Figure S16. STEM images of the sample and EDS mapping of the V signals. The STEM image of the sample during (a) the first cycle, (b) the second cycle and (c) the third cycle, and their corresponding EDS mapping of V. The white dotted lines represent the interface between Na_3VP and NZSP.

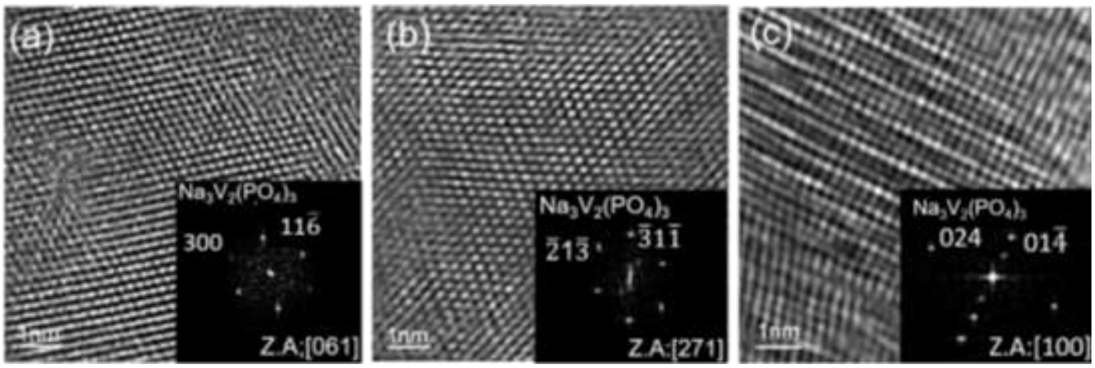

Figure S17. HRTEM images of cathode. Crystal structures of cathode after (a) the first cycle, (b) the second cycle and (c) the third cycle.

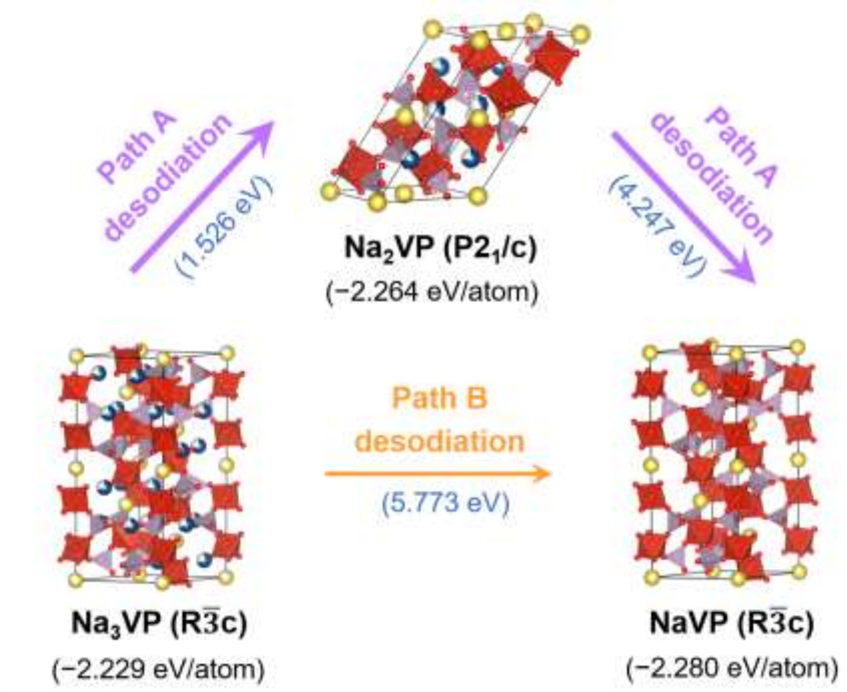

Figure S18. Schematic diagram illustrating the evolution of the Na_3VP crystal structure during the desodiation process, along with the corresponding formation energy (black digits) and the desodiation energy (blue digits).

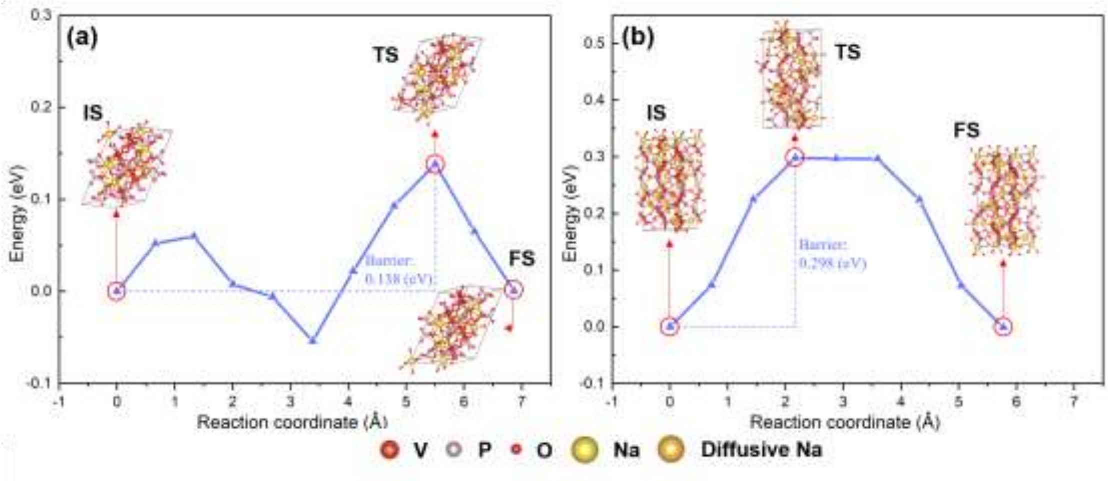

Figure S19. Migration energy profiles of the Na^+ ion in the (a) Na_2VP and (b) Na_3VP crystals, as well as the corresponding initial-state (IS), transition-state (TS), and final-state (FS) structures.

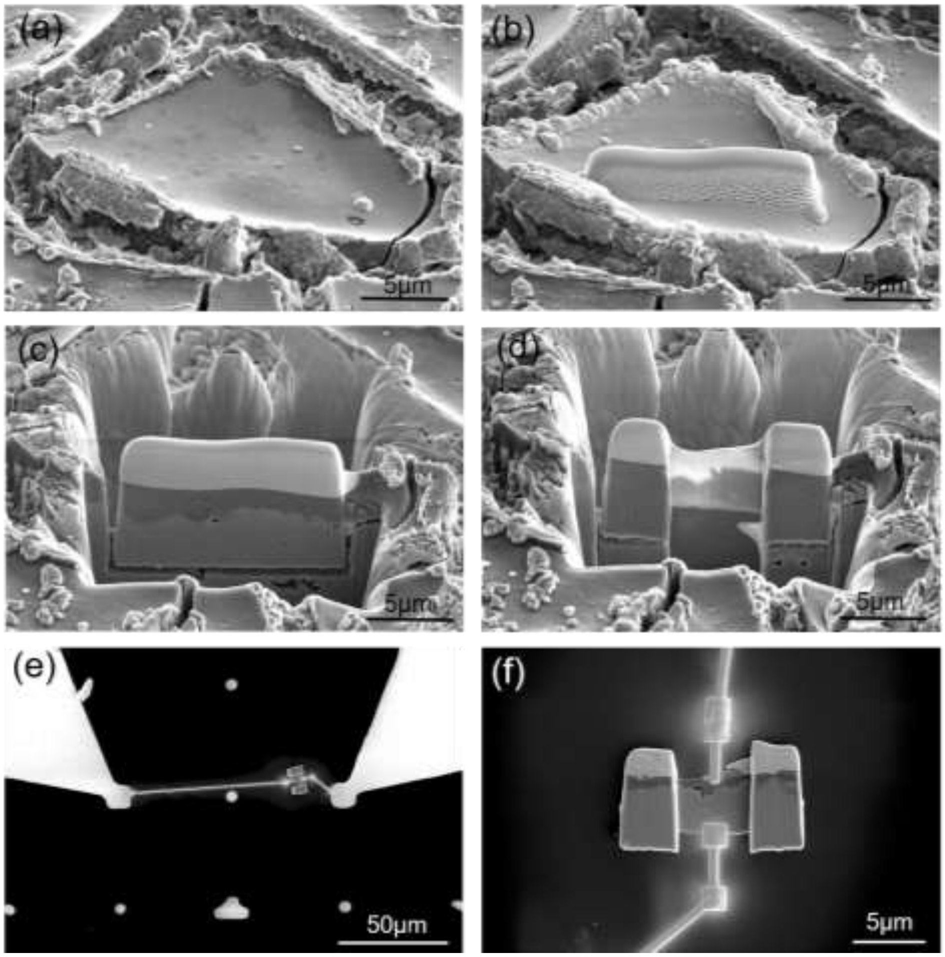

Figure S20. SEM images showing the preparation of the TEM sample and the deposition of electrodes with the FIB system. (a) The surface of the Na_3VP/NZSP sample. (b) Protective layer of Pt deposited on the sample surface. (c) Trenches milled on both sides of the TEM sample. (d) *In situ* TEM sample after the low-kV cleaning process. (e) Deposition of the TEM sample with Pt wires on the top and bottom sides of the sample. (f) An enlarged SEM image of the *in situ* TEM sample.

Table S1. Unit cell parameters, average V–O distances and V-O octahedron volumes for the compositions NaxVP, (x = 1, 2, and 3)

	space group	a (Å)	b (Å)	c (Å)	average V-O (Å)	V-O octahedron volume (Å^3)
Na_3VP ((Nano Energy 2019, 65, 104040))	$R\bar{3}c$	8.7321	8.7321	21.844	1.891	8.767
Na_2VP (Chem. Mater. 2022, 34, 1, 451–462)	$P2_1/c$	15.2377	8.6082	8.7391	1.994	10.186
NaVP (Chem. Mater. 2022, 34, 1, 451–462)	$R\bar{3}c$	8.42631	8.42631	21.4772	1.903	9.116
